# Probiotics for oral health: do they deliver what they promise?

**DOI:** 10.3389/fmicb.2023.1219692

**Published:** 2023-07-06

**Authors:** Wannes Van Holm, Katalina Lauwens, Pieter De Wever, Art Schuermans, Naiera Zayed, Ferda Pamuk, Mehraveh Saghi, Pedro Fardim, Kristel Bernaerts, Nico Boon, Wim Teughels

**Affiliations:** ^1^Department of Oral Health Sciences, University of Leuven KU Leuven, Leuven, Belgium; ^2^Centre for Microbial Ecology and Technology (CMET), Ghent University (UGent), Ghent, Belgium; ^3^Bio-and Chemical Systems Technology, Reactor Engineering and Safety, Department of Chemical Engineering, University of Leuven KU Leuven, Leuven, Belgium; ^4^Faculty of Medicine, KU Leuven, Leuven, Belgium; ^5^Faculty of Pharmacy, Menoufia University, Shebeen El-Kom, Egypt

**Keywords:** probiotic, lyophilization, freeze drying, viability, oral health, periodontitis, dental caries, adhesion

## Abstract

Probiotics have demonstrated oral health benefits by influencing the microbiome and the host. Although promising, their current use is potentially constrained by several restrictions. One such limiting factor lies in the prevailing preparation of a probiotic product. To commercialize the probiotic, a shelf stable product is achieved by temporarily inactivating the live probiotic through drying or freeze drying. Even though a lyophilized probiotic can be kept dormant for an extended period of time, their viability can be severely compromised, making their designation as probiotics questionable. Additionally, does the application of an inactive probiotic directly into the oral cavity make sense? While the dormancy may allow for survival on its way towards the gut, does it affect their capacity for oral colonisation? To evaluate this, 21 probiotic product for oral health were analysed for the number of viable (probiotic), culturable (CFU) and dead (postbiotic) cells, to verify whether the commercial products indeed contain what they proclaim. After isolating and uniformly lyophilizing three common probiotic species in a simple yet effective lyoprotective medium, the adhesion to saliva covered hydroxyapatite discs of lyophilized probiotics was compared to fresh or reactivated lyophilized probiotics. Unfortunately, many of the examined products failed to contain the claimed amounts of viable cells, but also the strains used were inadequately characterized and lacked clinical evidence for that unknown strain, questioning their label of a ‘probiotic’. Additionally, lyophilized probiotics demonstrated low adhesive capacity compared to their counterparts, prompting the question of why fresh or reactivated probiotics are not currently used.

## 1. Introduction

Probiotics, live microorganisms which when administered in adequate amounts confer a health benefit on the host (Hill et al., 2014), have shown benefits for a variety of oral diseases including periodontitis (Hardan et al., 2022; Zhang et al., 2022), tooth decay (Twetman, 2018), mucositis (Cereda et al., 2018), oral cancers (Sakiinah et al., 2022), pharyngotonsillitis (Wilcox et al., 2019), and oral candidiasis (Mundula et al., 2019). However, due to the patient and probiotic strain diversity, as well as poor quality of some studies, the benefits of probiotics for oral health are variable (Mundula et al., 2019; Sang-Ngoen et al., 2021). The use of ‘precision probiotics’, probiotics specifically chosen or even designed for a pathology, to address the heterogeneity inherent to probiotic strains, the hosts and their microbiomes, and high-quality research may be required to demonstrate their effectiveness (Veiga et al., 2020). In addition to selecting an ineffective probiotic strain, inadequate preparation of the probiotic product and insufficient time for reactivation and adhesion to oral surfaces may reduce the efficacy of probiotics for oral health. These interdependent factors are only partially determined by the biological properties of the probiotic. They are primarily influenced by how probiotic products are prepared. The first issue arises from the production of a shelf stable product. Producing a product containing living bacteria can be challenging since living cells require a continuous supply of fresh nutrients to survive or alternatively these living cells can be treated to enter a temporary dormant state. The latter can be accomplished through removal of all intracellular water, which results in a cessation of biological processes which is intended to be reversible. The most common procedure to accomplish this is through freeze-drying or lyophilization (Wolkers and Oldenhof, 2015; Kieps and Dembczynski, 2022). The ingenuity of this procedure lies in the inactivation of the bacteria through a freezing process as well as the use of the triple point of water, the temperature and pressure at which the three phases (gas, liquid, and solid) of water coexist in thermodynamic equilibrium, allowing for the sublimation and removal of the water molecules through a vacuum (Wolkers and Oldenhof, 2015).

If performed correctly, lyophilized bacteria maintain their cellular integrity while being metabolically inactive. These can then be kept dormant for an extended period of time and resume their activity when rehydrated (Broeckx et al., 2016). While straightforward for many species, sensitivity to the lyophilization process can differ drastically (Bircher et al., 2018; Phùng et al., 2022).

However, while lyophilization may be the most effective technique currently available for probiotic conservation, it is not infallible. The primary factors impeding its effectiveness include suboptimal lyoprotectants (agents aiding in freezing and/or storage), the conditions during lyophilization, and the storage of the lyophilized powder (Broeckx et al., 2016; Merivaara et al., 2021; Kieps and Dembczynski, 2022). The combination of these factors may result in a product that loses viable cells during or immediately following lyophilization, thereby changing the correct designation of some of the cells in the product from “probiotics” to “postbiotics” (Salminen et al., 2021). Inadequate lyophilization can be a significant problem limiting the efficacy of probiotics, despite their viability being one of the most basic qualifiers (Binda et al., 2020). The second problem occurs after lyophilization, just before the probiotic reaches its final destination. The lyophilized probiotic is designed to stay inactive until rehydration. However, both rehydration and subsequent reactivation of the probiotic can take some time.

This could be a significant issue for probiotics for oral health, which only reside in the oral cavity shortly. Before being swallowed, inactive probiotics may passively and reversibly adhere to the salivary pellicle, but temporary or even persistent colonisation requires an active effort from the probiotic to irreversibly adhere (Chua et al., 2020). If the duration of reactivation exceeds the time the probiotic spends in the oral cavity, the probiotic is unlikely to adhere adequately. Several studies have evaluated the oral adhesion of various live probiotic cultures which show extensive oral colonization (Haukioja et al., 2006; Ravn et al., 2012), but the majority of oral probiotics do not come as live cultures. The most common formulation for probiotics for oral health is lyophilized and compressed into a lozenge, which could diminish the probiotics’ adhesion potential and ultimately the amount of probiotics that the consumer receives. While ISO standards exist for evaluating (gastrointestinal) probiotic supplements (Binda et al., 2020), there are no guidelines or standards for determining an effective dose for probiotics for oral health that accounts for losses through washout. While the exact mechanisms are not fully understood, rehydrating the probiotic before administration is advantageous for probiotics for gastrointestinal (Arellano-ayala et al., 2021) and potentially oral health. Providing optimal reconstitution conditions may enhance their function (Muller et al., 2010; Broeckx et al., 2016) and possibly boost their adhesion potential.

In the present study, we evaluated the lyophilization-related issues of loss of viability and adhesive potential of the probiotics. First, commercially available products for oral health were analysed to determine whether they can be considered as genuine probiotics or should be classified a postbiotics, by considering if the contained microorganisms are either “live” (Hill et al., 2014) or “inanimate” (Salminen et al., 2021) respectively. Subsequently, isolates of commonly used probiotics (*Lacticaseibacillus paracasei*, *Limosilactobacillus reuteri*, and *Streptococcus salivarius*), were uniformly lyophilized, and evaluated for their adhesive potential to saliva coated hydroxyapatite discs.

## 2. Methods

### 2.1. Probiotic products tested

Probiotics recovered from products (Table 1) were cultured under anaerobic or aerobic (5% CO_2_) conditions on Man, Rogosa and Sharpe agar (MRS; Merck, Darmstadt, Germany) at 37°C. Products containing *Bacillus* species were additionally grown on LB agar (Merck) at 37°C and the product containing *K. marxianus* was grown on yeast extract peptone dextrose agar (YPD) at 30°C. Each product was also grown once on blood agar to ensure lack of major contaminants.

**Table 1:** Probiotic products evaluated.

Ranked product	Promised dose	Dose(s) at what time	Type	Probiotics in product^a^^,^^b^
1	1.00E+09	At time of manufacture + before expiration	Lozenge	Proprietary blend of *Lacticaseibacillus paracasei* (?), *Limosilactobacillus reuteri* (?), *Latilactobacillus sakei* (?), *Ligilactobacillus salivarius* (?)
2	5.00E+09	(?)	Lozenge	Proprietary blend of *Streptococcus salivarius* K12, *S. salivarius* M18, *Bacillus coagulans* (?)
3	(?)	(?)	Lozenge	Proprietary blend of *L. reuteri* (?), *S. salivarius* K12, *S. salivarius* M18
4	2.00E+08	Before expiration	Lozenge	*L. reuteri* ATCC PTA 5289, *L. reuteri* DSM 17938
5	3.00E+09	(?)	Lozenge	Proprietary blend of *Lactobacillus acidophilus* (?), *L. reuteri* (?), *L. paracasei* (?), *L. salivarius* (?), *Streptococcus thermophilus* (?), *S. salivarius* K12, *S. salivarius* M18
6	1.00E+08	(?)	Lozenge	Proprietary blend of *Streptococcus uberis* KJ2, *Streptococcus oralis* KJ3, *Streptococcus* rattus JH145
7	1.00E+09	(?)	Lozenge	*Kluveromyces marxianus fragilis* B0399, *Bifidobacterium lactis* HN019
8	1.00E+09	At time of manufacture	Lozenge	*S. salivarius* M18
9	5.00E+08	At time of manufacture	Lozenge	*S. salivarius* K12
10	7.00E+09	(?)	Lozenge	Proprietary blend of *L. paracasei* (?), *L. reuteri* (?), *L. salivarius* (?), *L. acidophilus* (?), *B. coagulans* (?)
11	2.00E+09	At time of manufacture + before expiration	Lozenge	Proprietary blend of *L. paracasei* 8,700:2, *Lactiplantibacillus plantarum* DSM 6595, *L. acidophilus* (?), *L. reuteri* (?), *S. salivarius* K12, *S. salivarius* M18
12	8.00E+09	(?)	Lozenge	*Weissella cibaria* CMU, *W. cibaria* CMS1
13	1.00E+09	(?)	Powder	Proprietary blend of *Lacticaseibacillus casei* (?), *L. acidophilus* (?), *L. salivarius* (?), *L. paracasei* (?), *Lacticaseibacillus rhamnosus* (?), *L. plantarum* (?), *B. lactis* (?), *Bifidobacterium bifidum* (?), *Bifidobacterium longum* (?), *Lactobacillus delbrueckii subsp. bulgaricus*, *Bifidobacterium breve* (?)
14	2.50E+09	At time of manufacture + effective level (?) before expiration	Lozenge	*S. salivarius* K12
15	5.00E+09	At time of manufacture + effective level (?) before expiration	Lozenge	*L. rhamnosus* GG
16	7.50E+08	At time of manufacture	Powder	Proprietary blend of *Bacillus subtilis* DE111, *B. coagulans* (?), *B. bifidum* (?), *L. rhamnosus* (?), *L. acidophilus* (?), *L. paracasei* (?)
17	1.00E+09	At time of manufacture + before expiration	Lozenge	Proprietary blend of *S. salivarius* K12, *S. salivarius* M18, *L. casei subsp. casei* (?), *L. paracasei* (?), *L. plantarum* (?), *L. reuteri* (?), *L. salivarius* (?), *B. lactis* BI-04, *L. rhamnosus* GG, *B. breve* (?), *Bifidobacterium infantis* (?), *S. thermophilus* St-21
18	3.00E+09	At time of manufacture	Lozenge	Proprietary blend of *B. coagulans* (?), *L. acidophilus* (?), *B. lactis* (?), *L. plantarum* (?), *L. rhamnosus* (?), *L. casei* (?), *L. salivarius* (?), *L. bulgaricus* (?), *B. breve* (?), *L. paracasei* (?), *L. lactis* (?), *S. termophilus* (?), *L. brevis* (?)
19	(?)	(?)	Lozenge	*L. reuteri* (?)
20	3.00E+09	(?)	Lozenge	*L. plantarum* L-137
21	1.67E+09	At time of manufacture	Powder	Proprietary blend of *S. salivarius* (?), *L. reuteri* (?), *L paracasei* (?)

a Products were ranked in descending order by live cells as determined by v-qPCR (Figure 1). Promised doses (CFU) were determined as the per unit dose of probiotic (a single lozenge or powder capsule). Several products instructed to consume multiple units to reach the advertised CFU dose, so the advertised dose was divided by that number to obtain the promised per unit dose. If a product disclosed a minimum dose before expiry, this was used as the promised dose instead of the dose at manufacturing.

b (?): Unknowns in promised dose, time of promised dose and strains used.

### 2.2. Enumeration of CFU, live and total cells in tested probiotic products

To mimic oral release of the probiotic, *in vitro* parameters were chosen to allow the product to dissolve and probiotic reconstitution before analysis (Table 2). Lozenges were aseptically crushed to a fine powder and all powders were transferred to 10 mL preheated phosphate buffered saline (PBS; considered as first dilution). After mixing by inverting, products were further dissolved by incubation for 15 min at 300 rpm. Upon completion of incubation, each product was vortexed for 15 s before creating a series of tenfold dilutions. Dilutions were plated on MRS agar by means of a spiral plater and grown for 48 h before enumerating colony forming units (**CFU**; only dilutions with 30–300 CFU/plate were used). The second dilution was used for enumeration of live (**v-qPCR**) and total (**qPCR**) cells according to Van Holm et al. (2021). Briefly, two samples were taken for DNA extraction. One sample was viability-treated with PMAxx (Biotium, Hayward, CA, United States) to eliminate qPCR detection of membrane-permeable/dead cells. The second, untreated sample was used to quantify total bacterial DNA (of both viable and dead cells). DNA was extracted with the QIAamp^®^ DNA Mini Kit (QIAGEN, Hilden, Germany) and analysed using qPCR with nonspecific primers for the 16S rRNA gene (forward: P338 5’-ACTCCTACGGGAGGCAGCAG-3′; and reverse: P518 5’-ATTACCGCGGCTGCTGG-3′). qPCR was performed with a CFX96 real-time system (Bio-Rad, Hercules, CA, United States) with reactions consisting of 12.5 mL of Takyon Rox SYBR master mix dTTP blue (Eurogentec, Seraing, Belgium), 1 mL of each primer at 300 mM final concentration (IDT, Haasrode, Belgium) and probe and 5.5 mL of Milli-Q water. Cycle conditions were: an initial step at 50°C for 2 min and 95°C for 10 min, followed by 45 cycles of 95°C for 15 s and 60°C for 1 min.

**Table 2:** Hypothetical comparison of parameters of probiotic release *in vivo* versus *in vitro* conditions.

Parameter	*In vivo*	*In vitro*
Time	5 min on average	15 min
Release from lozenge	Slowly by dissolving	Completely crushed tablet before adding to liquid
Volume (saliva/PBS)	1–5 mL	10 mL (mostly dissolved, except for large lozenges)
Salivary washout	Yes	No
Mixing with saliva/liquid	Mastication by tongue and teeth	Inverting, incubation at 300 rpm and 15 s vortex

### 2.3. Creating uniformly lyophilized probiotics of three commonly used probiotic species

To evaluate the effect of lyophilization on adhesion of the probiotics, subcultures were created from CFUs of three products containing the most commonly used probiotics: *L. paracasei* (from product 1 with primers from Kim et al. (2020), **no strain designation**), *L. reuteri* (**ATCC PTA 5289** from product 4), and *S. salivarius* (**K12** from product 9). Probiotics were grown in MRS broth for 18 h before centrifugation (5 min, 6000 × g) and washed once with PBS. Concentrations were adjusted to 1 × 10^9^ cells/mL and then concentrated and resuspended in lyoprotective medium. Bodzen et al. recently determined that the simple combination of 5% sucrose with 5% casein micelles is ideal for lactobacillus lyophilization and shelf-life (Bodzen et al., 2021). Forgoing the isolation of the casein micelles, skim milk powder (approximately 80% casein and 20% whey) was used as an easily accessible substitute. Based on this, four variations of lyoprotective media were tested: lyomedium 1: 10% (w/v) skim milk (Merck, Darmstadt, Germany), lyomedium 2: 10% (w/v) sucrose (Merck, Darmstadt, Germany), lyomedium 3: 5% skim milk with 5% sucrose, and lyomedium 4: 5% skim milk with 5% sucrose with 0.25% (w/v) ascorbate (Merck, Darmstadt, Germany) as an antioxidant (Wolkers and Oldenhof, 2015). Aliquots were made using 1.5 mL Eppendorf tubes and 5 mL glass HPLC vials (Avantor, Pennsylvania, United States), with each aliquot containing 1 × 10^9^ cells, drawn from 300 μL of lyoprotective medium.

Aliquots were snap frozen in liquid nitrogen before lyophilization in a LyoQuest (Telstar, Woerden, Netherlands) with a condenser trap set at −85°C and vacuum below 75 mtorr or 0.1 mbar. Aliquots were dry within 4 h, without additional shelf heating or secondary drying, even though these steps could further reduce water content for even longer shelf life. Aliquots were stored at room temperature and at 4°C.

Cell concentrations and viability were evaluated before and after lyophilization by flow cytometry (FCM) using a FACSVerse cytometer with a blue 488 nm laser and a red 640 nm laser (BD Biosciences, New Jersey, United States) as described previously (Zayed et al., 2023). Briefly, samples before and after lyophilization were diluted with PBS to 10^5^–10^6^ cells/mL and stained with SYBR^™^ Green I (1× final; Invitrogen, Thermo Fisher Scientific, Massachusetts, United States) and propidium iodide (4 μM final Thermo Fisher Scientific, Massachusetts, United States) and incubated for 20 min at 37°C. Forward scatter (FSC-A), side scatter (SSC-A), green fluorescence signals (FITC-A), and red fluorescence signals (PerCP-CY5.5-A) were used to characterize and gate live cells using the fresh cultures of the probiotics.

Before FCM evaluation, live probiotic cells were resuspended in lyoprotective medium to the same concentration as the lyophilized probiotic to remove influences of the medium as a confounding factor.

### 2.4. Adhesion of fresh versus lyophilized probiotics of three commonly used probiotic species

To mimic dentition, hydroxyapatite discs (D: 12.7 mm; h: 1 mm; Hitemco Medical, New York, United States) were coated with 1 mL filter sterilized saliva for 24 h at 37°C in a 24 well plate. Probiotic adhesion of *L. paracasei*, *L. reuteri*, and *S. salivarius* was evaluated by adding 1 mL of fresh or lyophilized probiotics (both resuspended and diluted in PBS at 2 × 10^8^ cells/mL) to the discs to ensure a final concentration of 1 × 10^8^ cells/mL. Per replicate, 10 discs were inoculated in parallel to allow for attachment during 5, 10, 15, 20, 30, 45, 60, 90, and 120 min, as well as 24 h, at 37°C and 300 rpm. At each timepoint, discs were dip-rinsed in 1 mL PBS before transferring to 1.5 mL of PBS after which adhered cells were recovered through 15 s vortexing. For 24 h, biofilms were chemically disrupted with trypsin for 45 min before vortexing. Cells recovered from discs were enumerated with FCM as described above.

### 2.5. Reactivation of lyophilized *Limosilactobacillus reuteri* and comparison of adhesive potential against lyophilized and fresh probiotic

Reactivation of a lyophilized probiotic, which was done for *L. reuteri* only, was performed by resuspending the powder in either MRS, M9 minimal medium (Difco, BD Biosciences, New Jersey, United States) with 2% w/v D-glucose (MM + gluc), or filter-sterilized saliva, followed by an incubation period of 2 or 24 h at 37°C before addition to discs. All reactivated probiotics were readjusted to 1 × 10^8^ cells/mL and added to saliva-covered discs for 30 min and compared to the fresh and non-reactivated lyophilized probiotic.

### 2.6. Statistical analysis

Statistical analysis was performed in R 4.2.0. Statistical differences between all conditions were all parametric and analysed with an ANOVA with Tukey HSD multiple comparisons (95% CI), but only a relevant selection was presented for readability.

## 3. Results

### 3.1. Selection of probiotic products and packaging information of the tested products

Nine European products (also available in the United States) and twelve American products (not available in the European Union) were purchased and summarized in Table 1.

Products were selected if they advertised oral health benefits (e.g., gingival inflammation, carious lesions, halitosis, tonsillitis, and thrush). Products were curated to select a wide variety of probiotic species, single species products, multispecies products, and high (>10^9^) or low (~10^8^) CFUs per dose. Many ‘duplicate’ products with the exact same composition were avoided, although their probiotic content may have differed due to differences in manufacturing. While fresh probiotic products (e.g., yogurts) were sought after, none for oral health were found on the Belgian, French and Dutch markets, although many were available for gut health.

Numerous products contained a wide variety of proprietary probiotic blends, probiotics with no strain designation, or strains with no clinical evidence of their effectiveness. Only five of the twenty-one products (products 4, 8, 9, 14, and 15) contained fully characterized and clinically supported probiotics for oral health. While diversity in available products was sought after, most probiotics were lactic acid bacteria, with only two *Bacillus* species and one yeast (*K. marxianus*) species being used. Aside from the products’ contents, the packaging information varied greatly between products. While eleven of the twenty-one products disclosed that the CFU on the label was at the time of production, ten of the products did not specify whether the CFU was at the time of production or was guaranteed to be present before the expiration date. Four of the twenty-one products indicated a guaranteed dose before the expiration date, and three of the products promised an “effective dose” before the expiration date, but did not disclose the exact number. Seventeen of the twenty-one products lacked clear information on the actual number of probiotics prior to expiration, assuming that the CFU determination was made at the time of production for products that do not indicate the time of CFUs. Twelve products contained a desiccant and one product packed in a glass container.

### 3.2. Enumeration of CFU, live and total cells recovered from probiotic products

The quantity of total, viable, and CFUs per single dose was assessed of the twenty-one products (Table 1). Products were ranked based on the average number of viable/live probiotics (yellow; Figure 1) as this the primary component of the probiotic, rather than the total number of cells (viable + non-viable; red) or the cultivability of the probiotic (CFU; blue). All products were examined at least one year prior to their expiration date, with some having as much as four years remaining.

**Figure 1:**
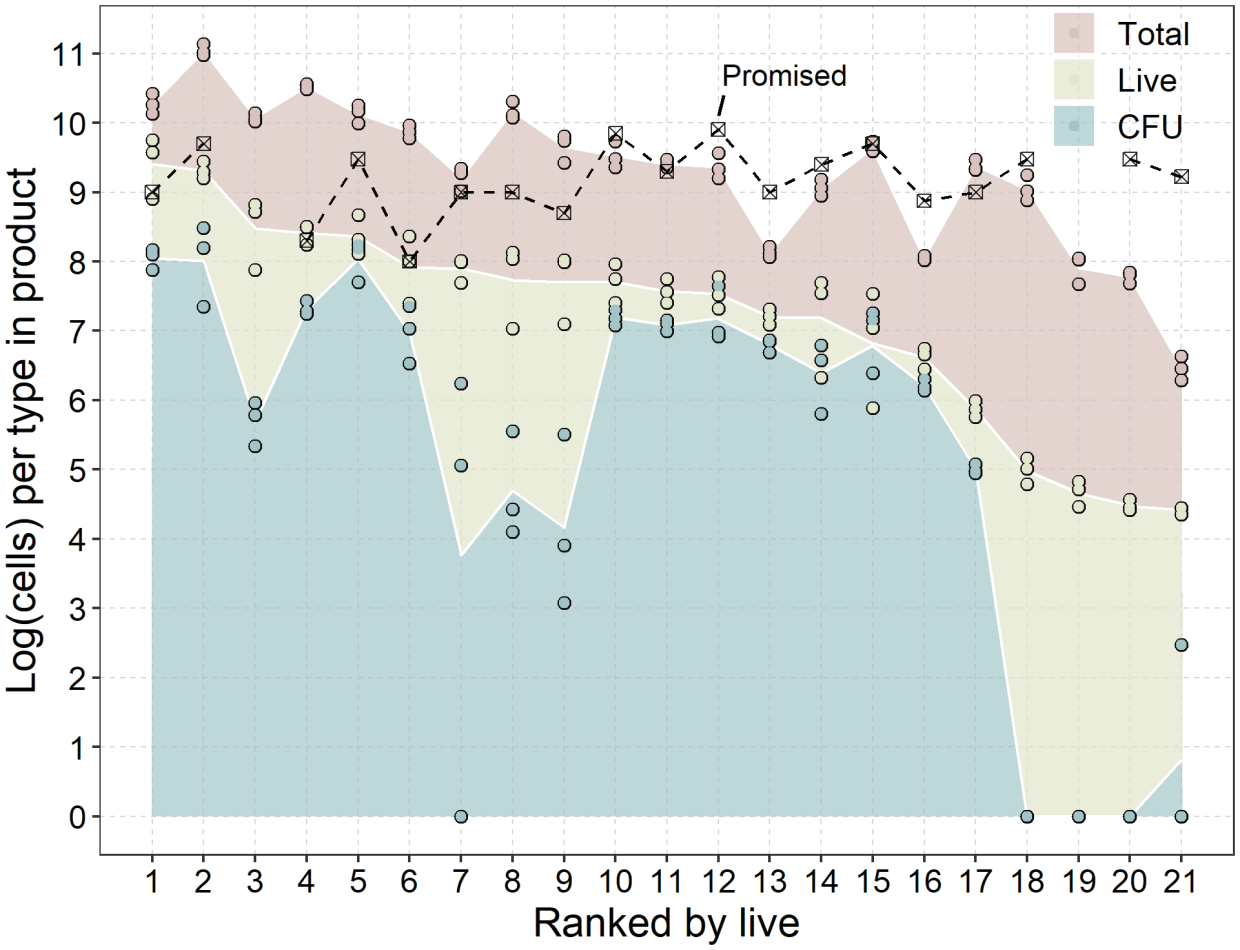
Promised dose, colony forming units (CFU), live-and total cells of each product. Products were ranked on the *x*-axis in descending order from the average of live or viable-but-not-culturable (VBNC) probiotics according to Table 1. Data are presented as the base ten logarithm of the number of cells recovered from the products (Table 1) and analysed through a dilutions series plated on MRS agar (CFU), viability qPCR (live) and regular qPCR (total) from replicates on separate days (*n* = 3). Promised doses are presented as the per unit CFU dose.

All of the examined products contained fewer CFUs of probiotics per dose than was advertised. This disparity could be the result of a suboptimal *in vitro* release of the probiotics in the tablets. However, the hypothetical comparison of *in vitro* and *in vivo* release conditions (Table 2), suggests that the *in vitro* releasing conditions were more lenient than the *in vivo* conditions.

Although the absolute number of CFUs may be underestimated, there were still distinct differences between the products tested. Although the products advertised CFUs within the range of 2 × 10^8^ to 7 × 10^9^ CFU/dose, the actual number of CFUs ranged from 0 to 10^8^ CFUs.

Detections of live or viable-but-not-culturable (VBNC) probiotics from the products were more in line with the promised dose, with four products closely meeting the advertised dose of probiotics (less than half a log difference). Four products had detections within 1 log of the advertised dose.

While both the total and CFU followed the reducing trend according to the live cells, several exceptions were observed. Several products (nr. 3, 7, 8, 9, 18, 19, 20, and 21) had a noticeably lower CFUs to live cells compared to other products. On the other hand, two products (nr. 13 and 16) had the closest groupings of live, CFU and total cells, having the best live/dead probiotic ratio. These differences indicate that some processes utilized by the manufacturers result in drastic differences in viability and cultivability of the probiotic.

### 3.3. Impact of lyophilization on probiotic viability

**Figure 2:**
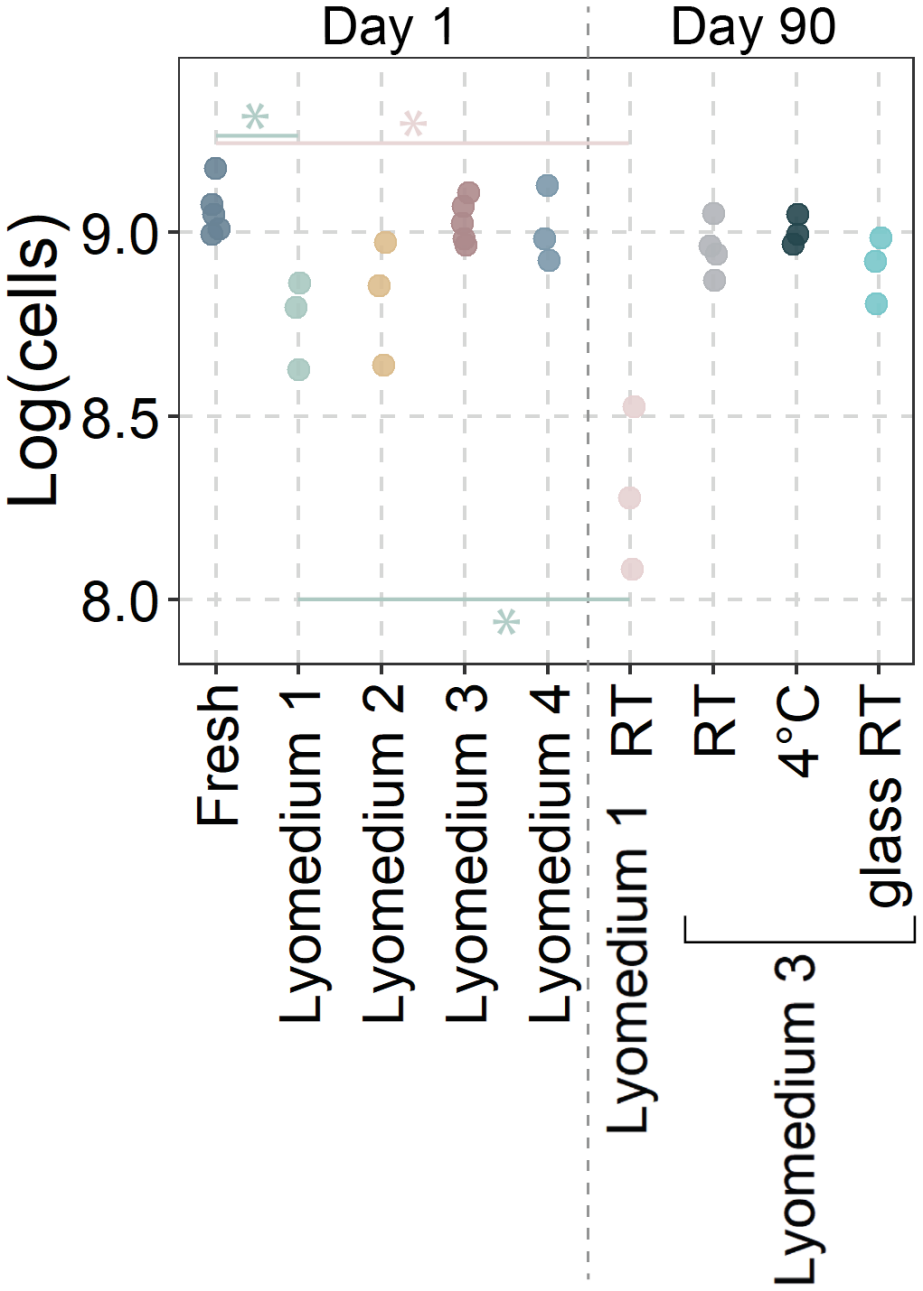
Viability of lyophilized *Limosilactobacillus reuteri*. Data represent the base ten logarithm of live cells in each sample as determined through flow cytometry. Lyophilization media consisted of Lyomedium 1: 10% skim milk; Lyomedium 2: 10% sucrose; Lyomedium 3: 5% skim milk and 5% sucrose; Lyomedium 4: 5% skim milk, 5% sucrose and 0.25% ascorbate. Lyomedium 1 and 3 were tested after 90 days at room temperature (RT) with lyomedium 3 also being prepared in a glass vial or storage in a 4°C fridge. Significant differences (*p* < 0.05; ANOVA with Tukey HSD; *n* ≥ 3) were only presented between the fresh probiotic and their respective lyophilized probiotic at day 90.

Due to the heterogeneity in size, composition and probiotic concentrations of the lozenges, a selection of the three most common probiotic species (*L. reuteri* in eight of the 21 tested products, *S. salivarius* in ten of the products and *L. paracasei* in eight of the products) were uniformly lyophilized for the next section. Short term viability of the lyophilized probiotics was assessed for *L. reuteri* (Figure 2).

While many types of lyoprotective media exist, four variations of basic components that are both inexpensive and commonly available were tested. One day after lyophilization, lyomedium 1 showed a significantly lower probiotic viability compared to the fresh probiotic (8.76 ± 0.1 versus 9.06 ± 0.06 log (cells/aliquot)). The viability in lyomedium 2 (8.82 ± 0.14) was also lower, but this difference was not statistically significant. The viability of probiotics in lyomedium 3 (9.03 ± 0.05) did not differ from that of fresh probiotics, and the addition of ascorbate (lyomedium 4; 9.01 ± 0.09) did not improve the probiotics’ short-term viability.

After 90 days, the viability of the probiotic in lyomedium 1 at room temperature decreased even further (8.3 ± 0.18), while the viability in lyomedium 3 remained high (8.96 ± 0.06), with no significant differences between room temperature, 4°C, plastic Eppendorf (9 ± 0.03), and glass vial storage (8.9 ± 0.07). Therefore lyomedium 3 was used to freeze-dry *S. salivarius* and *L. paracasei* in the subsequent experiment.

### 3.4. Adhesion of fresh versus lyophilized probiotics of three common probiotic species

**Figure 3:**
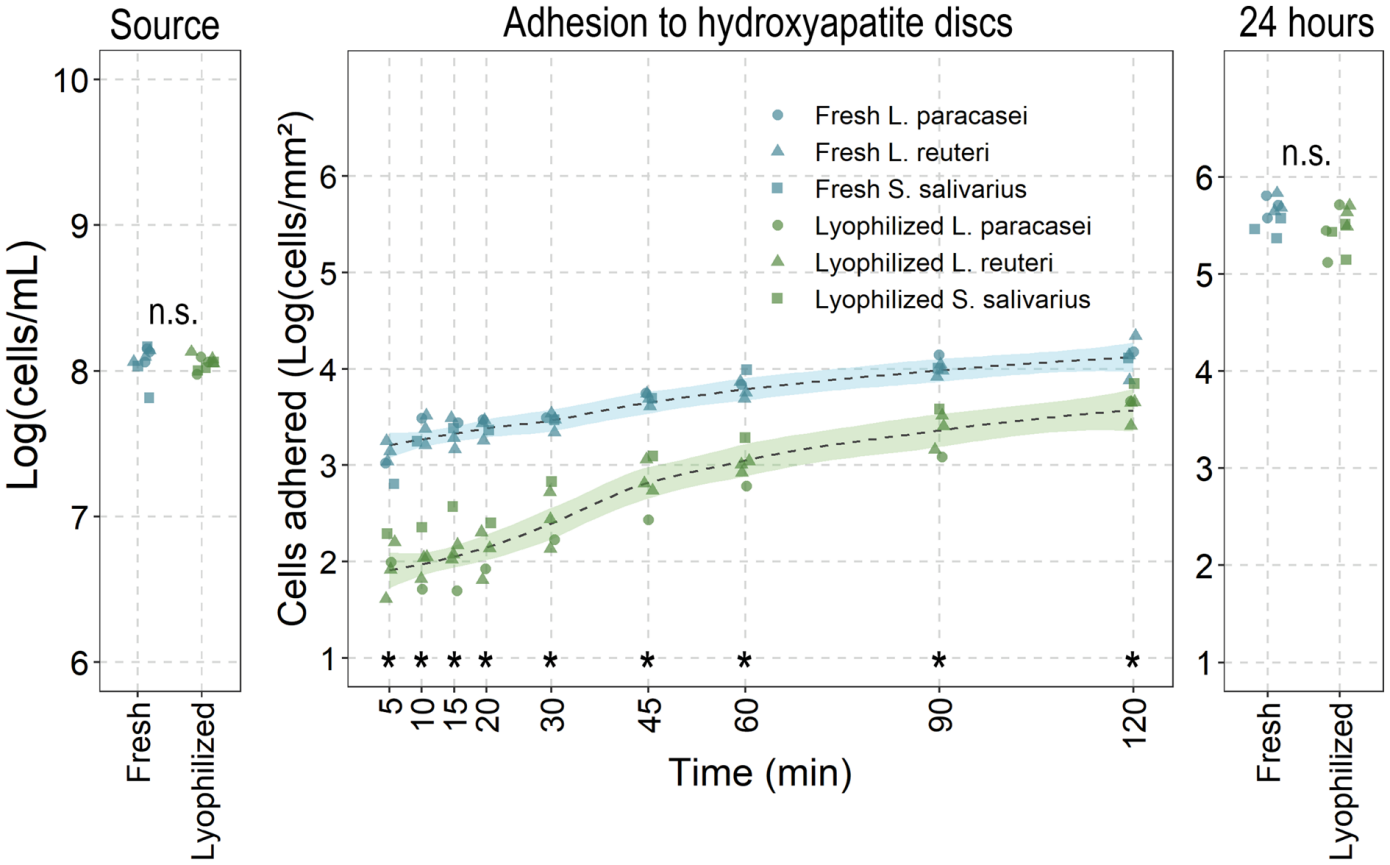
Fresh versus lyophilized probiotics’ adhesive-and biofilm potential. Fresh and lyophilized probiotics were resuspended in PBS and adjusted to 1 × 10^8^ cells/mL before adding to saliva covered hydroxyapatite discs for the noted times before recovering and enumerating attached cells through flow cytometry. Data presented as the base ten logarithm of events gated as the live probiotic per millilitre or per square millimetre. *: statistically significant and n.s.: non-significant differences (*p* < 0.05; ANOVA with Tukey HSD) between all lyophilized probiotics to their fresh counterparts at that timepoint (all species combined: *n* = 5 for adhesion; *n* = 9 for source and 24 h).

The adhesion of fresh and lyophilized probiotics to saliva-coated hydroxyapatite discs was evaluated to simulate *in vivo* conditions (Figure 3). In the first 2 h, fresh cultures significantly adhered better than lyophilized probiotics (fresh: from 3.05 ± 0.15 up to 4.13 log (cells/mm^2^) versus lyophilized: from 2 ± 0.24 up to 3.64 ± 0.14), despite the fact that the number of viable cells in the source was identical (8.09 ± 0.04 and 8.1 ± 0.04). After 24 h of growth and matrix development (thus considered as biofilms rather than adhered cells), lyophilized probiotics exhibited the same amount of biofilm formation as fresh cultures (5.61 ± 0.11 and 5.72 ± 0.1). Adhesive potential between the different probiotic species tested was similar.

While events (cells passing through the flow cytometer’s detector) were detected from the lyophilized conditions prior to 30 min, the event rate was too low to reliably provide reliable measurements of the attached cells (<200 events/cells per second). At 30 min, the event rates consistently exceeded 200 events per second, allowing for accurate quantification. For this reason, 30 min was chosen as the evaluation period for the reactivation of the probiotics.

### 3.5. Adhesion of a reactivated lyophilized versus lyophilized and fresh probiotic *Limosilactobacillus reuteri*

**Figure 4:**
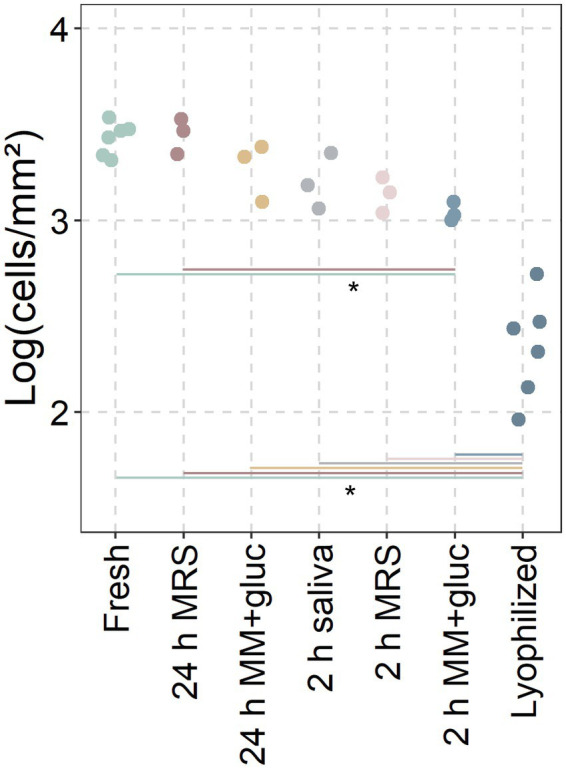
Adhesion of reactivated probiotics. Conditions between fresh cultures and lyophilized powders (only *L. reuteri*; incl. 3 replicates from Figure 2) were incubated for 2 or 24 h in MRS, Minimal Medium with glucose (MM + gluc) or saliva before adding to the discs. Adhered cells recovered from saliva covered hydroxyapatite discs after 30 min and analysed through flow cytometry. Data presented as the base ten logarithm of events gated as the live probiotic per square millimetre. *: statistically significant differences between conditions (*p* < 0.05; ANOVA with Tukey HSD; *n* ≥ 3).

Since the lyophilized probiotics showed significantly more adhesive potential after 2 and 24 h, the lyophilized *L. reuteri* were reactivated for 2 or 24 h in MRS, minimal medium with glucose or saliva (Figure 4). Reactivated *L. reuteri* showed significantly more adhesion than lyophilized probiotics (only 2.34 ± 0.24 cells/mm^2^) with only minor differences between reactivation medium and time (3.45 ± 0.08, 3.27 ± 0.12, 3.2 ± 0.12, 3.14 ± 0.08, and 3.04 ± 0.04 respectively). Only 2 h in minimal medium significantly differed from fresh cultures and 24 h of reactivation in MRS.

## 4. Discussion

While an expanding diversity of probiotics for oral health is being explored and developed into commercial products, many studies fail to account for other factors that may inhibit the probiotic’s optimal effectiveness. Using a systematic methodology and a cross-sectional sample of contemporary probiotics, the current study exposes some of these factors. This study identifies two essential elements that should be considered in any probiotic investigation: the actual dose of probiotics in a product and the effect of the product preparation on the adhesive potential of the probiotic.

### 4.1. What and how much do the probiotic products contain?

Several products did contain their advertised amounts as live or viable but not culturable (VBNC) cells. For eights of the products (nr. 3, 7, 8, 9, 18, 19, 20, and 21), the difference between CFU and VBNCs was significantly greater than for others, indicating that certain manufacturing procedures had a greater impact on cultivability than on viability. While these VBNCs may have lost their ability to grow on agar, VBNC probiotics have been shown to maintain metabolic activity and probiotic function (e.g., 16S rRNA integrity, reductase and esterase activity) (Lahtinen et al., 2008). Their presence may still provide the probiotic antimicrobial and immunoregulatory mechanisms for the remainder of their life cycle.

Furthermore, while these VBNCs may no longer be culturable on agar under normal conditions, other bacterial VBNCs have been found to regain their cultivability when exposed to specific resuscitation-promoting factors (Ramamurthy et al., 2014). Even while these factors are best understood for pathogens, it is likely that other micro-organisms have similar resuscitation mechanisms (Pan and Ren, 2023). Identifying these resuscitation-promoting factors for probiotics could innovate their lyophilized administration.

Despite the fact that fifteen of the products lacked the advertised number of living cells, they contained a substantial number of dead probiotics. Therefore they could rather be considered as a postbiotics (Salminen et al., 2021). While some evidence exists for the use of postbiotics for oral health (Ishikawa et al., 2020; Mendonça Moraes et al., 2022), their effects will always remain transient as they are even more unlikely to incorporate into the oral microbiome than their live counterparts. However, if the primary mode of action is retained after death, postbiotics may be preferable to probiotics due to their longer shelf life and likely lack of side effects due to its transient nature (Zolkiewicz et al., 2020). Unfortunately both the use and regulation of postbiotics are still in their infancy (Vinderola et al., 2022), especially for use in the oral cavity. However, none of the products assessed in this study were promoted as postbiotics, but rather as probiotics.

While this was not the primary objective of this study, 11 of the products (nr. 1, 3, 5, 10, 11, 13, 16, 17, 18, 19 and 21) lacked clear information on which probiotic strains were included. Without any strain information, the used strain naturally did not have one positive human clinical trial supporting the probiotic effects of that specific strain. Sixteen of the products did not meet with these two criteria of Binda et al.’s interpreted definition of probiotics (Binda et al., 2020). Another noteworthy observation is that most probiotic products contained mostly lactic acid bacteria. There might be potential for other genera such as *Rothia* species that show probiotic potential (Rosier et al., 2020), but are unfortunately not yet on the market.

Lyophilization, a common method for preserving bacteria, requires that the bacteria be suspended in a medium that aids in maintaining their viability during freezing, water removal, and storage. Due to the nature of lyophilization, loss of viable cells can be anticipated. Therefore, it is preferable for a probiotic product to include information about the number of viable microorganisms. In fact, most products (*n* = 17/21) lacked information on the number of living microorganisms before the expiry date. This is in line with probiotic products for gastrointestinal and respiratory health (Fredua-Agyeman et al., 2016; Zawistowska-rojek et al., 2016; Zamfir et al., 2023). While several products disclosed a CFU dose at the manufacturing stage, it is often unclear if this quantification was performed before or after lyophilization. Moreover, as shown, depending on the lyoprotectant medium and storage conditions, additional viability can be lost during storage. This can lead to significant variations in remaining viability over time. Only four of the products (products 1, 4, 11, and 17) disclosed an actual guaranteed dose before the expiry date, highlighting the need for more detailed information and transparency by the manufacturer.

Since little information was disclosed about the products, the products were dissolved and examined for CFUs as well as living and total cells. Even though the expiry dates were always more than a year out, fifteen of the examined products did not contain the specified doses of living cells, and none provided the declared number of CFUs. While these discrepancies may be partially attributable to the generic *in vitro* release method of the probiotic, which may not be the most appropriate for some of the evaluated probiotics, it can be anticipated that the oral cavity would likely receive an even lesser quantity of living probiotics. Despite the possibility of underestimation and despite receiving the same treatment, significant differences between products were identified. While the promised CFUs per dose were roughly within a 1.5 Log range (from 2 × 10^8^ up to 7 × 10^9^ CFU per dose), the viable, total and actual CFUs differed several Log values between products, ranging from close to the expected dose to no CFU detected. This is in line with previous research identifying major differences in the quality of various probiotic products with drastic differences in CFUs per dose of commercial products (Zawistowska-rojek et al., 2016). These differences could be attributable to the probiotic’s sensitivity to lyophilization, growth medium and conditions, lyoprotective medium, lyophilization procedure, and storage conditions (Broeckx et al., 2016). Due to the proprietary nature of these factors, it is impossible to determine what does and what does not work. Nevertheless, 11 of these products did not meet the requirements to qualify as probiotics on the basis of containing sufficient live cells (at least 10^8^ viable cells) (Binda et al., 2020), although this number is based on efficacious doses for gastro-intestinal probiotics. Products containing *Streptococcus* probiotics may have contained more CFU than detected since they might consist of chains of cocci (as their genus name implies), potentially lowering the CFU counts as a chain would have likely formed a single colony. Although the CFU determination by the manufacturer also would encounter this underestimation.

Unfortunately, the effective probiotic dose for oral health is currently unknown. Similar to gastrointestinal probiotics, the effective dose of probiotics for oral health may be strain-dependent. However, it can be hypothesised that the adhesive potential may also influence the effective dose. A formulation of a probiotic that is more adhesive will likely require a lower concentration to achieve an effective dose. This is especially important for probiotics for oral health since the amount of time that anything, including a probiotic, remains in the oral cavity is significantly shorter than in the gastro-intestinal tract, which may result in that some probiotics being able to colonize the gut (Alander et al., 1999), but not the oral cavity (Alander et al., 1999; Yli-Knuuttila et al., 2006).

### 4.2. Towards better product preparation and storage

Extensive research has been conducted to determine which lyoprotectants could be used for optimal lyophilization of probiotics. Combinations of sugars (lactose or sucrose) and dairy-derived products (skim milk, casein, or whey) have been utilized as lyoprotectants for a very long time due to their accessibility, affordability, and adequate protective properties (Kieps and Dembczynski, 2022). Since many of the tested products disclosed that they contain dairy, dairy derivatives were likely used as lyoprotectants. As demonstrated by the present study and others (Bodzen et al., 2021), substituting a portion of the skim milk/casein with the relatively inexpensive sucrose significantly enhanced the viability of the probiotics. Requirements of lyoprotective medium may be species-and strain dependent, but the used medium worked well for the tested probiotics at a very low cost.

In addition, unfavourable culturing conditions prior to lyophilization can affect the viability of the probiotics (Kieps and Dembczynski, 2022). These culturing conditions were not disclosed in the tested products and may have contributed to the observed discrepancies between the advertised CFU’s and the actual viable cells.

It is well known that aside from growth and lyophilization, storage conditions of the probiotic product can affect the viability of the probiotics. Storing probiotic products at 4°C can drastically improve shelf life (Abe et al., 2009; Hirsch et al., 2021; Kiani et al., 2021). Only one of the products disclosed that refrigeration could extend shelf life. In the current study, 4°C did not improve the preservation of viability over a 3 month period as there was no significant difference with the viability of the lyophilized probiotics that were stored at room temperature. The data also showed that the lyomedium is a more important factor for maintaining viability than temperature in the short term. These data do not rule out that temperature may play a more important role when maintaining probiotic products in the long term.

Reducing humidity, thereby preventing preterm rehydration, is also known to improve the viability of the probiotics (Wolkers and Oldenhof, 2015). This can be achieved by including a desiccant or using storage containers with a low water permeability such as glass. Twelve products did contain a desiccant and only one was packed in a glass container. These products will likely have a reduced rehydration during storage, resulting in a longer shelf life. In the current study, glass vials did not show an improvement in viability after 3 months over plastic tubes at room temperature suggesting that this factor also has limited effect in the short term.

### 4.3. Probiotic adhesion to saliva coated hydroxyapatite of three common probiotic species

In addition to lyophilization limiting the probiotic’s viability, it can be hypothesized that the primary concept of this process, the inactivation of the probiotic, can also affect the probiotic’s oral colonisation.

Due to the risk that the heterogeneity of the products could skew the results, three of the most common species (*L. paracasei*, *L. reuteri*, and *S. salivarius*) were isolated and lyophilized in the same simple lyoprotective medium containing identical amounts of live cells. Their adhesion on saliva-coated hydroxyapatite discs was significantly lower than that of their living counterparts for the first 30 min. Given that oral lozenges are unlikely to remain in the mouth for more than 5 min, the probiotics are likely already on their way to the gastrointestinal tract as opposed to remaining in the oral cavity. The salivary flow and subsequent washout of the probiotics raises the question of whether these lozenges are the optimal delivery method and whether they can offer an effective probiotic dose.

A potential solution could be rehydrating and reactivating the lyophilized probiotic before oral administration. Rehydration conditions have long been known to affect the viability of lyophilized bacteria (Font De Valdez et al., 1985), however little is known about their impact on probiotic activity (Broeckx et al., 2016). However, recent evidence coming from the gastrointestinal field suggests that both function and adhesion can be enhanced by prior rehydration (Arellano-ayala et al., 2021).

In the current study, when both the fresh-and lyophilized probiotics were incubated on the discs for 24 h, their adhesion/biofilm formation was nearly equal, indicating that time is a significant factor for their colonisation. While the time in the oral cavity may be limited, more time could be given to the probiotic to reactivate before oral administration. As a proof of concept, providing the lyophilized *L. reuteri* probiotic with 24 h of reactivation in MRS or minimal medium with glucose restored its adhesive potential. While long reactivation may yield optimal adhesion, this should be limited to avoid growth of unwanted contaminants. Reactivation of the probiotic for only 2 h already increased its adherence significantly, with only minor differences in which medium the probiotic was reactivated. While 2 h in MRS was sufficient for this *L. reuteri* strain to regain its adhesive potential, different probiotic species and strains may have different lyophilization and reactivation requirements, which should be assessed strain-by-strain. Adhesive potential may also be species and strain specific, which should also be considered when determining the required probiotic dose.

One of the products (product 5) recommended dissolving it in liquid, but did not indicate for how long. Despite the benefit of reactivation seen in the current study, this does not seem common practice for commercial probiotics for oral health.

### 4.4. Limitations of the current study

This study has a number of limitations. For example, only the *in vitro* adhesion to saliva-coated hydroxyapatite, as a proxy for teeth, was examined. The probiotics’ adherence to oral soft tissues, to which they may attach better, was not evaluated due to lack of a suitable soft tissue model. However, for the probiotic to have a direct effect on cariogenic and periodontal biofilms, it must adhere to the hard dental surfaces. However, a reactivated probiotic will likely also colonize soft tissues better, which may serve as a source of probiotic diffusion or indirect/proximal probiotic impact on the oral ecology. To confirm if reactivated probiotics are superior to their non-reactivated lyophilized form, additional clinical trials are necessary. Additionally, the properties of the strains to function as a probiotic (e.g., antimicrobial and anti-inflammatory effects) were not evaluated in this study. Especially anti-inflammatory probiotic effectivity perhaps does not require the probiotic to be present in the oral cavity as there is potential for systemic effects (Deandra et al., 2023), although the delivery methods of the tested products are designed for oral delivery rather than getting the probiotic to the gut to then act systemically.

Differences in probiotic efficacies may result in differences in needed probiotic doses. A final limitation is that the manufacturers of the products were not contacted to reveal their, most likely proprietary, production processes to evaluate differences between the products.

## 5. Conclusion

In summary, the analysis of some commercially available probiotics for oral health revealed two major issues. Firstly, many of the products do not fit the criteria as a probiotic supplement based on either being insufficiently characterized, not supported by clinical evidence or not containing live probiotics throughout their shelf life. Additionally, while lyophilized probiotics allow for optimal shelf life, their adhesive potential is limited in comparison to their fresh or reactivated counterparts.

Based on all of the above, it can be recommended that, firstly, there is currently no standard on what an effective dose is for probiotics for oral health. Especially considering that their adhesion is limited due to salivary washout, a new consensus developed by a panel of experts on a minimal adherent probiotic dose is vital.

Secondly, an improvement of the provided information is necessary. Too little products accurately disclose which probiotic they contained and how ‘alive’ the probiotic would actually be when consumed. While this can be improved by optimizing growth and lyophilisation, these factors were unknown for the products. Perhaps reducing the expiry date of some products from an absurd 4+ years to a shorter, yet more accurate date is desirable. Inclusion of a ‘Best by date’ or ‘Guaranteed CFU before expiration’ that accurately reflects the probiotic dose can already be a tremendous benefit. A large number of CFUs at an unknown time does not necessarily make it a better product.

Thirdly, considering the need of adherent probiotics could also benefit innovations in the probiotic delivery through reactivation or other delivery modalities to ensure optimal colonisation and thus probiotic efficacy.

Addressing these will result in better informed consumers, superior products and a better understanding of the efficacy and requirements of probiotics for oral health.

## Data availability statement

The raw data supporting the conclusions of this article will be made available by the authors, without undue reservation.

## Author contributions

WH, KL, WT, KB, and NB: conceptualisation of the project. WH, KL, AS, and FP: product analysis. WH, PW, PF, NZ, and MS: lyophilization and adhesion experiments. WH, KL, and WT: drafting manuscript. WH, KL, PW, AS, NZ, FP, MS, PF, KB, NB, and WT: critical analysis of data and review of manuscript. All authors contributed to the article and approved the submitted version.

## Funding

This study was supported by a grant from the Research Foundation – Flanders (Fonds Wetenschappelijk Onderzoek; FWO; G091218N) and a grant from the KU Leuven (C24/17/086).

## Conflict of interest

WT has lectured for BioGaia, one of the anonymized products. The KU Leuven has received a research grant and has conducted several clinical studies for BioGaia.

The remaining authors declare that the research was conducted in the absence of any commercial or financial relationships that could be construed as a potential conflict of interest.

## Publisher’s note

All claims expressed in this article are solely those of the authors and do not necessarily represent those of their affiliated organizations, or those of the publisher, the editors and the reviewers. Any product that may be evaluated in this article, or claim that may be made by its manufacturer, is not guaranteed or endorsed by the publisher.
